# Participation of Increased Circulating MAIT Cells in Lung Cancer: a Pilot Study

**DOI:** 10.7150/jca.69415

**Published:** 2022-03-06

**Authors:** Qian Zhang, Pengli Li, Wenqin Zhou, Shu Fang, Jiong Wang

**Affiliations:** 1 Department of Geriatric Respiratory and Critical Care, Anhui Geriatric Institute, the First Affiliated Hospital of Anhui Medical University, Hefei, Anhui, China; 2 School of Biomedical Engineering, Anhui Medical University, Hefei, Anhui, China; 3 3D-Printing and Tissue Engineering Center, Anhui Medical University, Hefei, Anhui, China

**Keywords:** Lung cancer, Peripheral blood, MAIT cell, CD38

## Abstract

Mucosal-associated invariant T (MAIT) cells are a subset of innate-like T cells that regulate the immune response via rapidly releasing inflammatory factors, including during progression of some tumors. However, the immunological role of MAIT cells is still unclear in lung cancer. We measured percentage, partial function, and clinical correlation of circulating MAIT cells from lung cancer patients through flow cytometry. Lung cancer patients displayed a high concentration of CD4^+^, CD8^+^, and activated CD38^+^CD8^+^MAIT cells, and a decrease of PD1^+^ double negative (DN) MAIT cells in peripheral blood. Meanwhile, increased levels of interferon-γ, interleukin (IL)-6, and 8 were examined in the serum of lung cancer patients. Importantly, we discovered a statistically positive association between accumulation of CD38^+^CD8^+^MAIT cells and reduced progression-free survival of lung cancer patients. While preliminary, the altering frequency of MAIT cells might be involved in dysfunctional immune response in lung cancer.

## Introduction

Lung cancer (LC) is one of the most common causes of solid tumor mortality around the world 1. Due to its often-advanced stage at detection and poor prognosis, lung cancer presents a considerable burden on human health globally 2. Lung cancers arise from the malignant lesions of the bronchial mucosa or glands and are divided into non-small cell lung cancer and small cell lung cancer based on histopathological classification 3. However, the immunologic mechanism underlying lung cancer remains controversial. Previous reports demonstrated that a suppressed immune response caused by increasing levels of myeloid-derived suppressor cells, M2 macrophages and regulatory T cells have a strong effect on evading host immunosurveillance and thus can promote lung cancer progression 4-14. In addition, there is mounting evidence proving the importance of participation of CD8^+^ or CD4^+^ T cells and natural killer cells in lung cancer progression 15-18.

Mucosal-associated invariant T (MAIT) cells are a subset of innate-like T cells that are widely distributed in peripheral blood (PB) and mucosal tissues 19-23. This unconventional T cell produces the semi-invariant T cell receptor, consisting of α chains (Vα7.2-Jα33) and β chains (Vβ2, 13, and 22) 21. MAIT cells are positively immunophenotyped as CD161^+^, CD3^+^ and T-cell receptor (TCR) Vα7.2^+^ cells and recognize microbial antigens of vitamin B2 presented by major histocompatibility complex-related protein 1 21-25. Emgård and his colleagues demonstrated that MAIT cells mainly contribute to the early immune response in the streptococcal toxic shock syndrome caused by group A streptococci 26. In addition, Proinflammatory factor signals can activate MAIT cells independent of TCR recognition, such as interleukin (IL)-12 or 18. 27-29. Activated MAIT cells will immediately release interferon (IFN)-γ and perforin during inflammatory responses 30-31. Recent evidence has emphasized the important role of MAIT cells in various microbial infection diseases (mycobacterium tuberculosis, hepatitis B and influenza) 32-35.

Tumors frequently occur in human mucosal layers like bronchial mucosa, where MAIT cells are enriched 19. Furthermore, the mucosal immune barrier can be destroyed by lung cancer due to the lesion of immune cells 16. Numerous previous studies have confirmed that frequencies of MAIT cell from patients were related to the occurrence, development, and prognosis of mucosal-associated cancers, such as gastric, colorectal, and esophageal cancer 36-39. Like other invariant natural killer T cells, MAIT cells can play a part in antitumor immunosurveillance via producing tumor-killing cytokine like tumor necrosis factor (TNF)-α in the cancer progression 40. For example, the circulating MAIT cells were found by Rodin et al to play a crucial part in the cancer cell elimination through infiltrating colon mucosa 38. However, higher production of IL-17A-related MAIT cell in the tumor microenvironment is closely linked with decreasing progression-free survival (PFS) 41. Thus, MAIT cell can have different immune effects in various malignancies, and the function and distribution of MAIT cell are still unclear in lung cancer.

In this work, we analyzed the production of serum cytokine and circulating MAIT cells in lung cancer and explored their plausible relevance to the prognosis of patients.

## Materials and Methods

### Patient samples

We collected heparinized peripheral blood from 35 primary LC patients hospitalized at the First Affiliated Hospital of Anhui Medical University and 35 age-matched healthy donors (HD). Otherwise, eligible LC patients with immunodeficiency, autoimmune disorders or immunotherapies were removed. None of the patients were given any medicine before sample collection. Secondary lung cancer or other malignancies were also included as exclusion criteria. All the patients' clinical data are listed in Table 1. Written informed consents were obtained from all human subjects under the ethical approval in this study (No. 20210418). Peripheral blood mononuclear cells (PBMCs) were isolated through density gradient centrifugation following the Ficoll-Paque (GE Healthcare, Sweden) protocol. The serum obtained after centrifugation was stored at -80 °C, and the PBMCs adjusted to 10^7^ cells before being stored in a cryopreservation medium (10% Dimethyl Sulphoxide and 30% fetal calf serum) at -80 °C.

### ELISA assay

Levels of IL-6, 8, 12, 17, 18 and IFN-γ in serum of all participants were evaluated by ELISA kits (Multisciences Biotech, Hangzhou, China) following the manufacturer's instruction.

### Flow cytometry

Frozen PBMC samples were processed into single-cell suspensions (1×10^7^/mL) after thawing in a 37 °C incubator and washing with phosphate-buffered saline. Cells were preincubated with FcR Blocking Reagent (Biolegend, USA) for 20 min at 4 °C before the following human monoclonal antibodies staining for 30 min at 4 °C: CD8 PerCP-Cy5.5, CD4 FITC, CD3 APC-Cy7, CD161 APC, CD38 PE-Cy7, CD279 (PD-1) PE-Cy7, TCR Vα7.2 PE (BD Biosciences: #560662, #555346, #557832, #550968, #560677, #561272 and Biolegend: #351706). Data were obtained using FACSVerse Flow Cytometer (BD Biosciences) after fluorescent compensation and analyzed using FlowJo_V10 software. Cutoff for positive cell staining was determined by fluorescence minus one (FMO).

### Statistical analysis

Statistical data were assessed using the GraphPad Prism 8.0 software. Differences between two groups were evaluated with unpaired two-tailed t tests. The linear association between variables was analyzed using Pearson's test with the Bonferroni correction. Kaplan-Meier analysis was performed for estimating PFS of LC patients. Log rank testing was implemented to weigh the survival curve. P-value less than 0.05 was considered as statistical difference.

## Results

### Distribution of different circulating MAIT cell subsets from patients with lung cancer and controls

**Figure 1:**
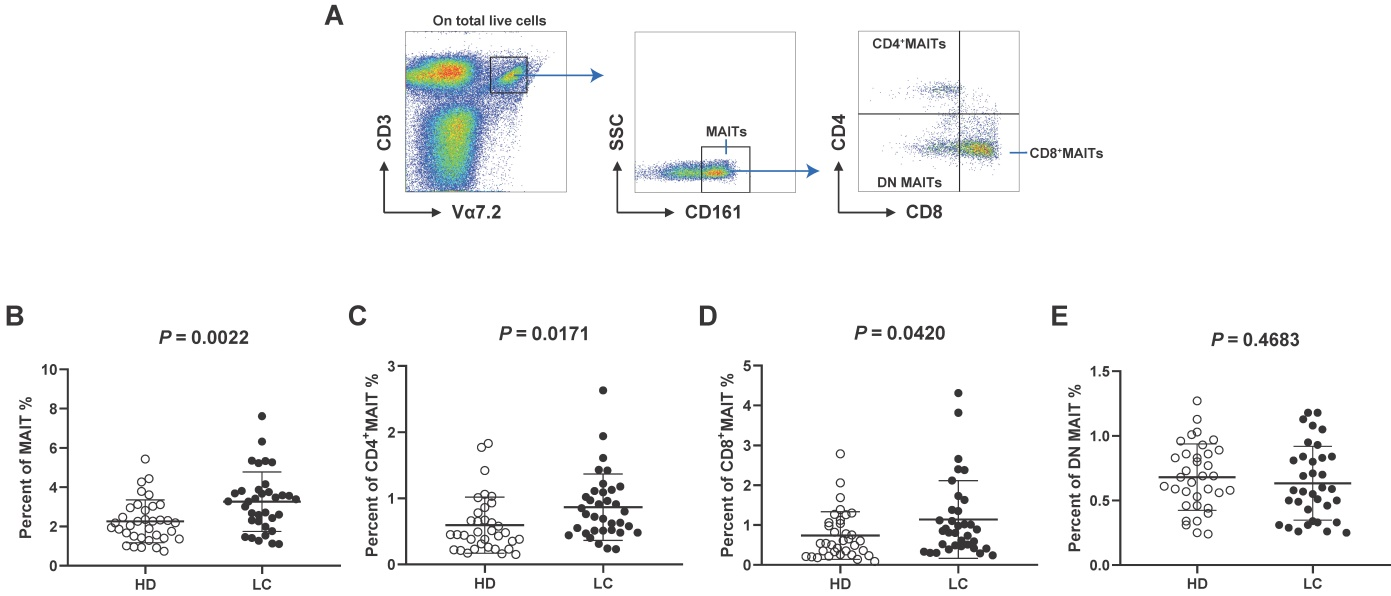
** Distribution of PB MAIT cell from LC patients and HD.** PBMC samples were collected from 35 HD and 35 LC patients and incubated with CD3, Vα7.2, CD161, CD4 and CD8 markers. First, MAIT cells were gated on CD3^+^Vα7.2^+^CD161^+^ cells, and then were categorized as CD4^+^, CD8^+^, and DN MAIT cells (A). The difference in frequencies of MAIT, CD4^+^, CD8^+^, and DN MAIT cell between LC patients and HD (B-E). P < 0.05 is regarded as significant.

We analyzed the distribution of circulating MAIT cell subsets from LC patients and HD. MAIT cells were characterized as CD3^+^Vα7.2^+^ CD161^+^cell via flow cytometry. We separated them into three subsets: CD4^+^, CD8^+^, and CD4^-^CD8^-^ (double negative, DN) cells by the expression of CD8 and CD4 (Fig. 1A). Our results displayed remarkably elevated frequencies of MAIT cell in PB of patients with LC compared to HD (Fig. 1B). In addition, LC patients showed elevated percentages of circulating CD4^+^ and CD8^+^MAIT cell (Fig. 1C, D). However, no other significant differences were measured between LC patients and HD in the frequencies of PB DN MAIT cell (Fig. 1E).

### Expression of CD38 and PD1 on circulating MAIT cell from all subjects

**Figure 2:**
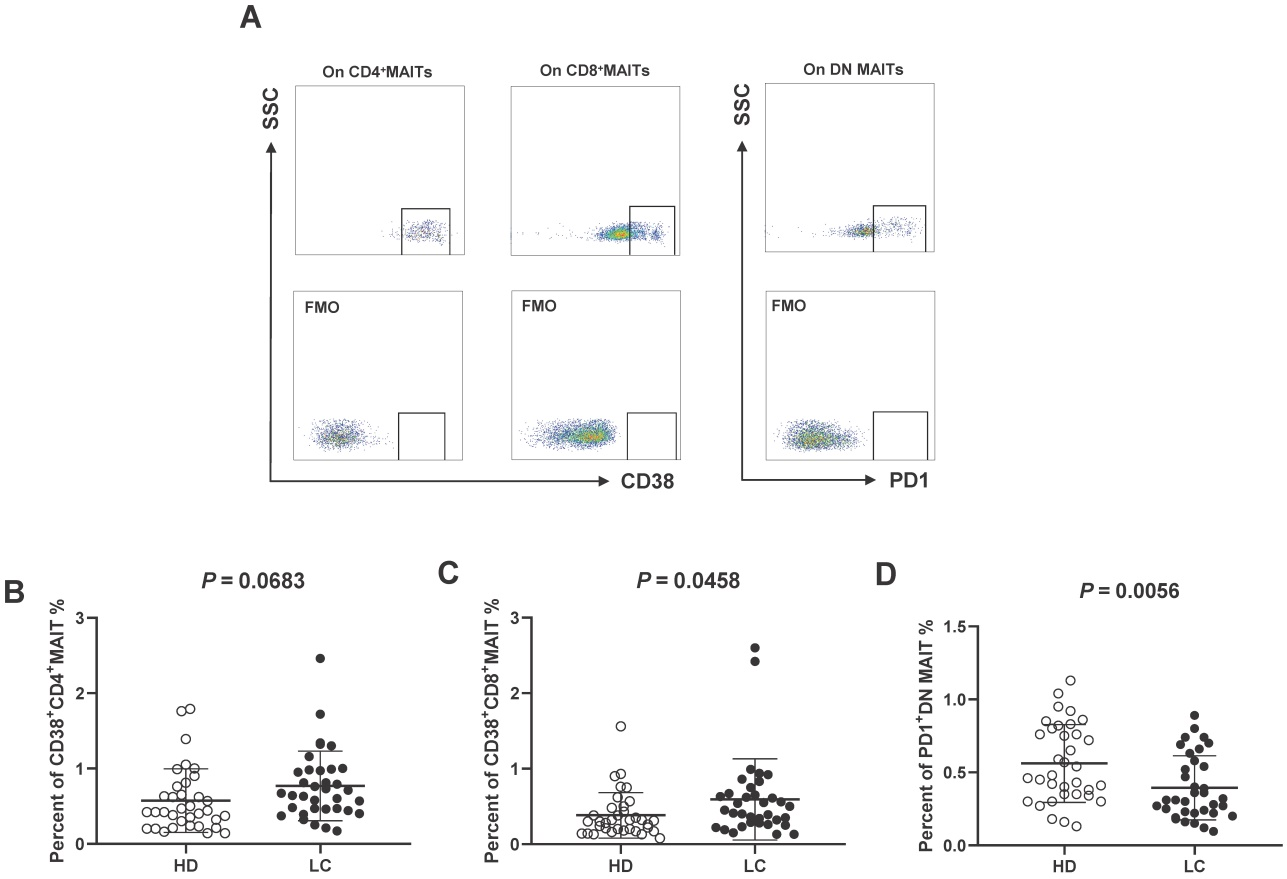
** CD38 and PD1 phenotypes of PB MAIT cell from LC patients and HD.** CD38^+^ and PD1^+^ cells were gated on CD4^+^, CD8^+^, and DN MAIT cells according to FMO controls (A). The Difference in percentages of CD38^+^CD4^+^, CD38^+^CD8^+^ and PD1^+^DN MAIT cell between LC patients and HD (B-E). P < 0.05 is regarded as significant.

Next, we investigated the activities of MAIT cells by evaluating CD38 (activation) and PD1 (dysfunction) levels according to FMO controls (Fig. 2A). No accumulation of CD38^+^CD4^+^MAIT cells was examined in the PB of LC patients (Fig. 2B), while we detected elevated percentages of CD38^+^CD8^+^MAIT cell (Fig. 2C). The levels of circulating PD1^+^DN MAIT cell from HD were significantly higher than LC patients (Fig. 2C, E).

### Enhancement of cytokines IL-6, 8/IFN-γ in serum of participants

**Figure 3:**
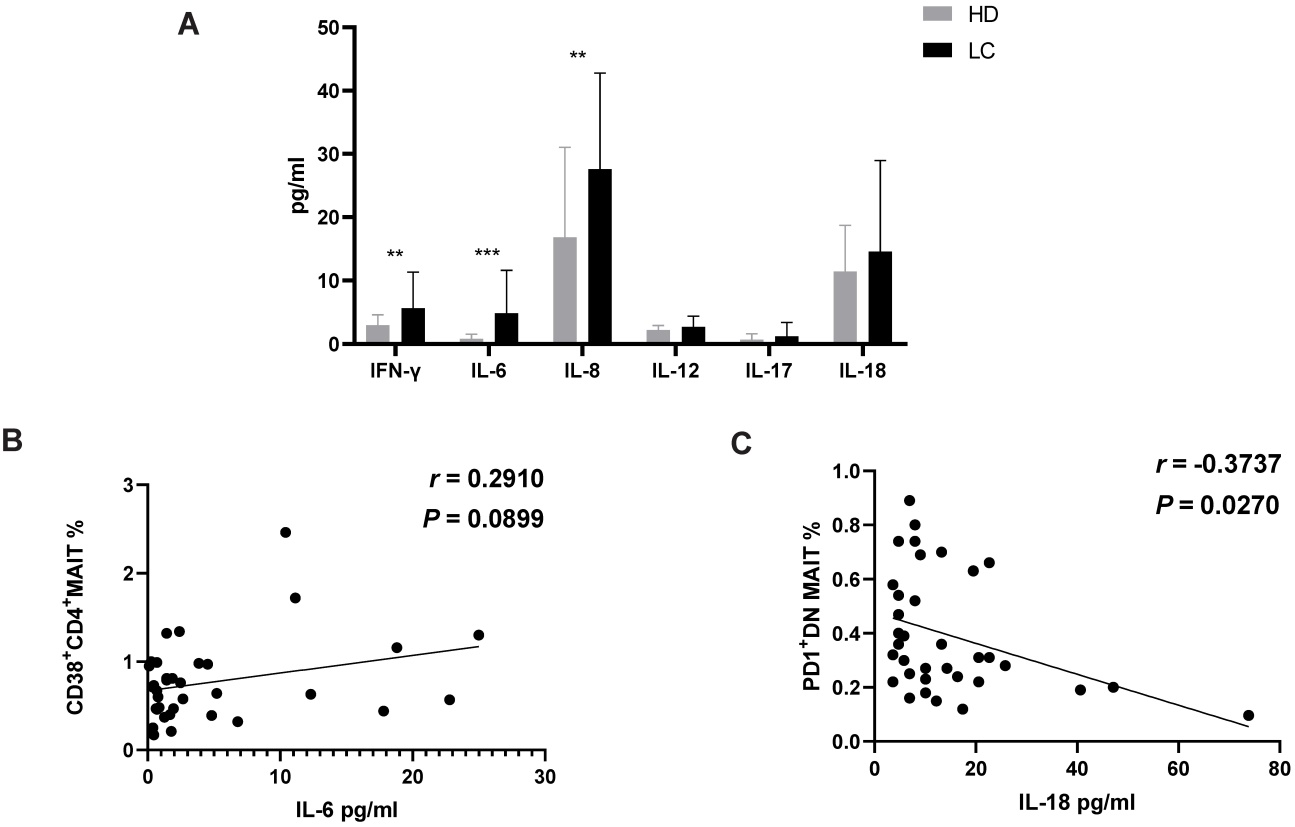
** Level of cytokines in serum of HD and LC patients, and the correlation between IL-6, 18 and circulating MAIT cells of LC patients.** IFN-γ, IL-6, 8, 12, 17 and 18 were detected in serum of HD and LC patients (A). The association between production of IL-6 and CD38^+^CD4^+^MAIT cell (B). The association between production of IL-18 and PD1^+^DN MAIT cell (C). ** displays P < 0.01 and *** displays P < 0.001. P < 0.05 is regarded as significant.

To demonstrate the function of MAIT cell-associated cytokines in lung cancer progression, these factors were measured in the serum from all participants. The results revealed a significant enrichment of cytokines IL-6, 8 and IFN-γ in serum of LC patients compared to HD. However, no significant differences were examined in serum IL-12, 17 and 18 (Fig. 3A). Additionally, levels of IL-6 showed a positive association with percentage of CD38^+^CD4^+^MIAT cell in the PB of LC patients (Fig. 3B). In contrast, a negative relationship between the production of IL-18 and percentage of PD1^+^DN MAIT cell was observed in LC patients (Fig. 3C).

### Association between circulating MAIT cell and progression-free survival of patients with lung cancer

**Figure 4:**
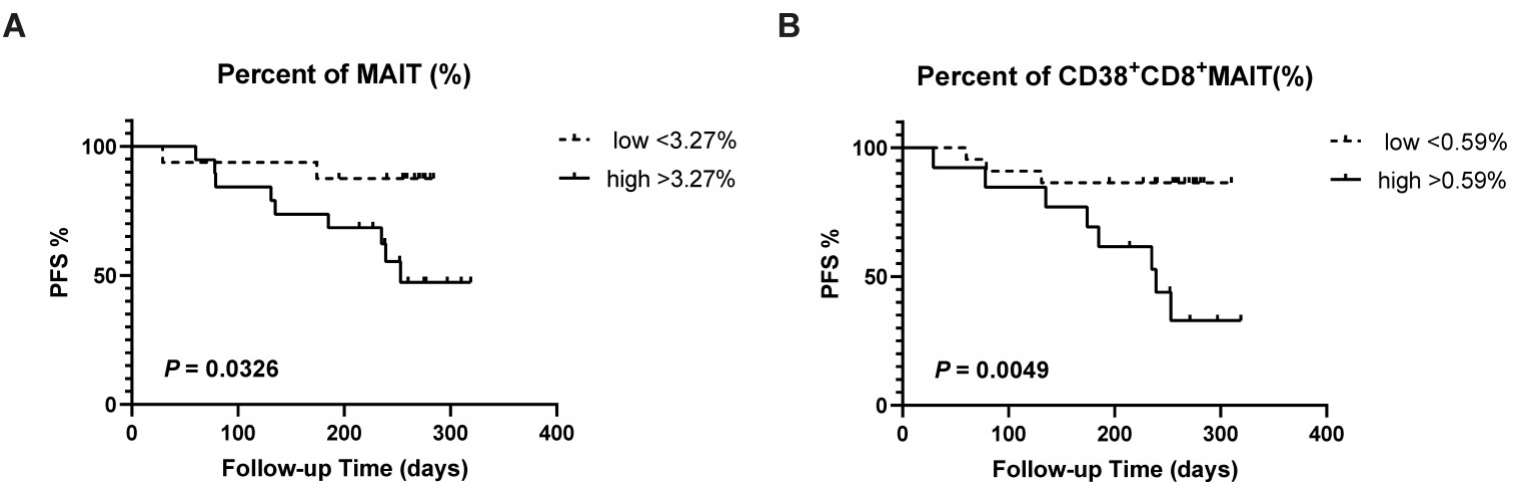
**Survival analysis of LC patients between percentages of CD38^+^CD8^+^MAIT cell and PFS.** The survival curve of low and high percentages from MAIT cell in the lung cancer patients across 320 days (A). The survival curve of low and high percentages from CD38^+^CD8^+^MAIT cell in the lung cancer patients across 320 days (B). P < 0.05 is regarded as significant.

LC patients were divided into low and high groups according to the mean percentage of immune cells. We detected a remarkably positive association between lower frequencies of circulating MAIT cell (>3.27%) and PFS (P = 0.0326; Fig. 4A). Meanwhile, a similar correlation was examined between a low level of circulating CD38^+^CD8^+^MAIT cell (>0.59%) and PFS (P = 0.0049; Fig. 4B).

## Discussion

As a kind of innate-like T cell, MAIT cell, like other CD8^+^ and CD4^+^ T cell, is detected at high levels in the immune microenvironment of malignant tumors 36, 41. Previous reports have shown that the tissues of gastric, colorectal, and esophageal cancer may be broadly infiltrated by MAIT cells, indicating these cells could participate in the pathogenesis of cancers 37-39. However, variable percentages of circulating MAIT cells are observed in various tumors. In this work, we explored the distribution, function, and clinical association of PB MAIT cell from patients with LC. We reveal that changes in levels of different MAIT cell types could be engaged in the immunological mechanisms underlying LC.

MAIT cells are categorized into 3 groups: CD4^+^, CD8^+^ and CD8^-^CD4^-^ (DN) cells as per expression of phenotype markers CD4 and CD8. Among them, CD8^+^MAIT cells are the most abundant and CD4^+^ the least abundant in human PB 42. But the distribution of these cell subsets in PB of LC patients is still unclear. Our results suggest an elevation of total MAIT cells in LC patients compared to HD. These data support the view that MAIT cell plays a key part in the immunological pathogenesis of lung cancer. More specifically, the level of CD4^+^ and CD8^+^MAIT cell was increased in LC patients, while no other significant differences were determined between frequencies of DN MAIT cell from two groups. This indicates that high level of CD4^+^ and CD8^+^MAIT cell might be involved in immune dysfunction during LC.

Previous reports demonstrated numerous phenotypic markers can be expressed on the MAIT cell surface, including CD38 and PD1 receptors. CD38 receptor expression represents cell activation, while PD1 exhibits cell exhaustion and dysfunction 34. We also measured the expression of CD38 and PD1 on circulating MAIT cells. Data reveal an apparent elevation of CD38^+^CD8^+^MAIT cell from patients with LC compared HD. However, the percentages of PD1^+^DN MAIT cells from LC patients were significantly lower than in HD. Dias et al revealed that DN MAIT subset might be derived from major CD8^+^MAIT subset, but also indicated different functions 42. This might underlie differences if the frequencies of these cells in LC patients. We hypothesize that these two MAIT cell subsets have opposing effects on the host immune reaction during LC.

Interestingly, MAIT cells in PB can rapidly secrete IFN-γ in response to tumor invasion, being directly activated with IL-12 and IL-18 28, 30. We detected a marked accumulation of IFN-γ, IL-6 and IL-8 in the serum of LC patients compared to HD. Moreover, we found a trend of positive correlation between serum IL-6 and circulating CD38^+^CD4^+^MAIT cells, which suggests an immunosuppressive function for this MAIT cell subset. In contrast, IL-18 negatively correlated with PD1^+^DN MAIT cells. Accounting for the previously evidenced tumorigenic properties of IL-6 and IL-18, we therefore hypothesize that CD38^+^CD4^+^MAIT and PD1^+^DN MAIT cell have distinct functions. This information is important in the characterization of the immune response and immunosuppression of LC patients.

Finally, numerous studies demonstrated that MAIT cells had an important effect on clinical prognosis of tumors 36-39. We examined the relationship between relative percentages of MAIT cell and PFS of LC patients to explore the predictive value of MAIT cell abundances in LC. A lower level of MAIT cell, and more specifically CD38^+^CD8^+^MAIT cell, was associated with improved PFS of LC patients, suggesting a detrimental effect increased to their abundance.

To conclude, the above data suggest that MAIT cells might generate and recruit inflammatory factors such as IL-6, 8, and IFN-γ in the immune response during LC, as suggested by their elevated production in LC patients. Meanwhile, tumor-infiltrating activity might be facilitated in the tumor microenvironment by elevated circulating CD8^+^ and CD4^+^ MAIT cells during LC. An elevated level of PB CD38^+^CD8^+^ MAIT cells were associated with reduced PFS, indicating an active role in lung cancer. Nevertheless, our analysis of these immune cells in PB is limited. It will be important to analyze immune response in the tumor microenvironment directly in future studies, which will help to elucidate LC mechanisms and generate new immunological therapies.

## Figures and Tables

**Table 1:** Clinical variables of subjects.

Characteristics	LC (n=35)	HD (n=35)		P-value	
Age, mean ± SD	57.69 ± 10.59	57.94 ± 6.29	0.9020		
Gender, n (%)					
male	21 (60.0%)	21 (60.0%)			
female	14 (40.0%)	14 (40.0%)			
Pathology, n (%)					
adenocarcinoma	22 (62.9%)				
squamous cell carcinoma	9 (25.7%)				
small cell lung cancer	4 (11.4%)				
Stage, n (%)					
I	6 (17.2%)				
II	0 (0.0%)				
III	4 (11.4%)				
IV	25 (71.4%)				
					

Abbreviation: LC, lung cancer; HD, healthy donator.

## Abbreviations

DN: double negative
FMO: fluorescence minus one
LC: lung cancer
PB: peripheral blood
